# Loss and Return of Sensation After Axillary Brachial Plexus Nerve Block: Distally or Proximally?

**DOI:** 10.1213/ANE.0000000000008175

**Published:** 2026-07-23

**Authors:** Hagen Bomberg, David Lorenzana, Neeti Sharma, Corsin Poltera, Urs Eichenberger

**Affiliations:** From the 1 Department of Anaesthesiology, Intensive Care Medicine and Pain Medicine, Balgrist University Hospital, Zurich, Switzerland; 2 Department of Outcomes Research Consortium, UTHealth, Houston, Texas.

The blockade of the axillary brachial plexus is an established anesthetic technique for surgeries on the upper extremities.^1,2^ However, the relationship between the return of sensation and the anatomical region remains unclear. We hypothesize that the loss and return of sensation after the axillary brachial plexus nerve block will first occur in the proximal part of the upper arm and last in the distal part.

## METHODS

Between March and August 2024, a prospective study was conducted with 50 patients. The study was registered (registration number NCT06443879) and ethically approved (Kantonalen Ethikkommission Zurich; BASEG-Nr. 2024-00012). Written informed consent was obtained from all participants.

Inclusion criteria were small surgery on the hand and forearm (ie, removal of radial osteosynthetic material), age ≥18years, and no allergy to mepivacaine.

All patients received a standardized 1% mepivacaine blockade of the axillary brachial plexus with ultrasound guidance and nerve stimulation monitoring for anesthesia. The needle was inserted in-plane from a posterior approach and advanced anteriorly to block the radial and ulnar nerves, and subsequently from an anterior approach directed posteriorly to block the musculocutaneous and median nerves. The mean amount of local anesthetic was 379 ± 49 mg. During surgery, all patients had spontaneous respiration without any airway device and were arousable.

The loss and return of sensation were assessed in various anatomical regions: distal humerus, elbow, proximal forearm, distal forearm, hand, and fingers. Sensory changes were tested using cold perception.^3^ The professional staff was trained and used a standardized documentation form with specific instructions (Supplemental Digital Content, S1, https://links.lww.com/AA/F837). Loss was tested every 5 minutes starting immediately after completion of the block. Return was tested at least every 20 minutes until full sensation was restored. Data integrity was evaluated according to specific rules to delete erroneously entered data and cases with missing information (proof of plausibility; Supplemental Digital Content, S2, https://links.lww.com/AA/F837).

The times for the loss and return of sensation were compared using a paired *t* test. Spearman correlation coefficients were used to test for correlation (SPSS Statistics 29). Continuous variables are expressed as means ± standard deviations.

## RESULTS

The final study population comprised 49 patients, because one patient had to be excluded (missing information about the return of sensation). The average age was 43 ± 18 years, with a body mass index of 24 ± 4 kg/m^2^ and an American Society of Anesthesiologists physical status classification of 1.9 ± 0.6. The patient population was evenly distributed between females (n = 24) and males (n = 25). All blocks were successful and no patient required an overnight stay (this also includes the one patient with missing data).

Loss of sensation in the fingers occurred significantly later than in the upper arm. That was true for the first sign (anterior: upper arm 4.8 ± 1.5 minutes vs fingers 7.0 ± 3.4 minutes, *P* = .003; posterior: upper arm 5.2 ± 2.8 minutes vs fingers 6.9 ± 3.4 minutes, *P* = .01; Figure 1A) and the complete loss of sensation/onset time (anterior: upper arm 11.9 ± 5.4 minutes vs fingers 17.1 ± 6.9 minutes, *P* < .001; posterior: upper arm 12.5 ± 6.7 minutes vs fingers 17.0 ± 8 minutes, *P* = .01).

**Figure 1.**
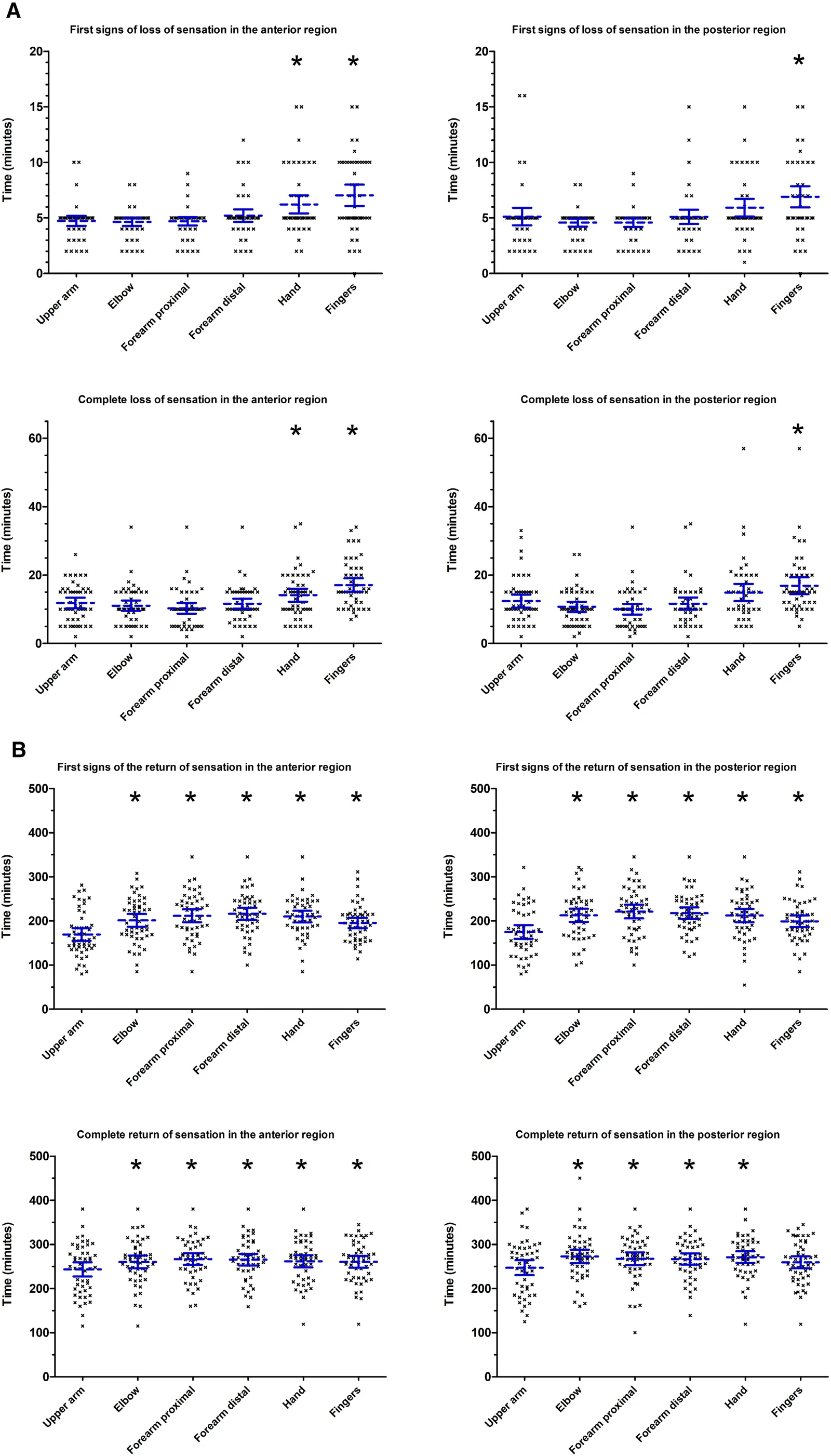
All patients (n = 49). Scatter dot plot with mean (blue short dash) and 95% confidence interval (blue line). *P < .05 versus upper arm. Two-sided *P* values <.05 were considered statistically significant. A, Loss of sensation (minutes). Loss of sensation was tested by the anesthesia staff in the operating room at least every 5 min until total loss of sensation of the arm. The first signs of loss of sensation: Time between the end of block placement and the first signs of loss of sensation. Complete loss of sensation = onset time: Time between the end of block placement and the complete loss of sensation. B, Return of sensation (minutes). Return of sensation was tested by the daycare nurses at least every 20 min until total return of sensation of the arm. The first sign of the return of sensation: Time between the end of block placement and the first sign of the return of sensation. Complete return of sensation = block duration: Time between the end of block placement and the complete return of sensation.

The initial return of sensation to the upper arm occurred significantly earlier than in the elbow, forearm, or hand (anterior: upper arm 170 ± 51 minutes vs hand 210 ± 45 minutes, *P* < .001; posterior: upper arm 177 ± 54 minutes vs hand 212 ± 52 minutes, *P* < .001) (Figure 1B). This was also evident in the complete return of sensation/block duration (anterior: upper arm 245 ± 56 minutes vs hand 262 ± 48 minutes, *P* = .01; posterior: upper arm 249 ± 59 minutes vs hand 271 ± 46 minutes, *P* = .001). The return of sensation in the elbow, forearm, and hand was comparable.

**Figure 2.**
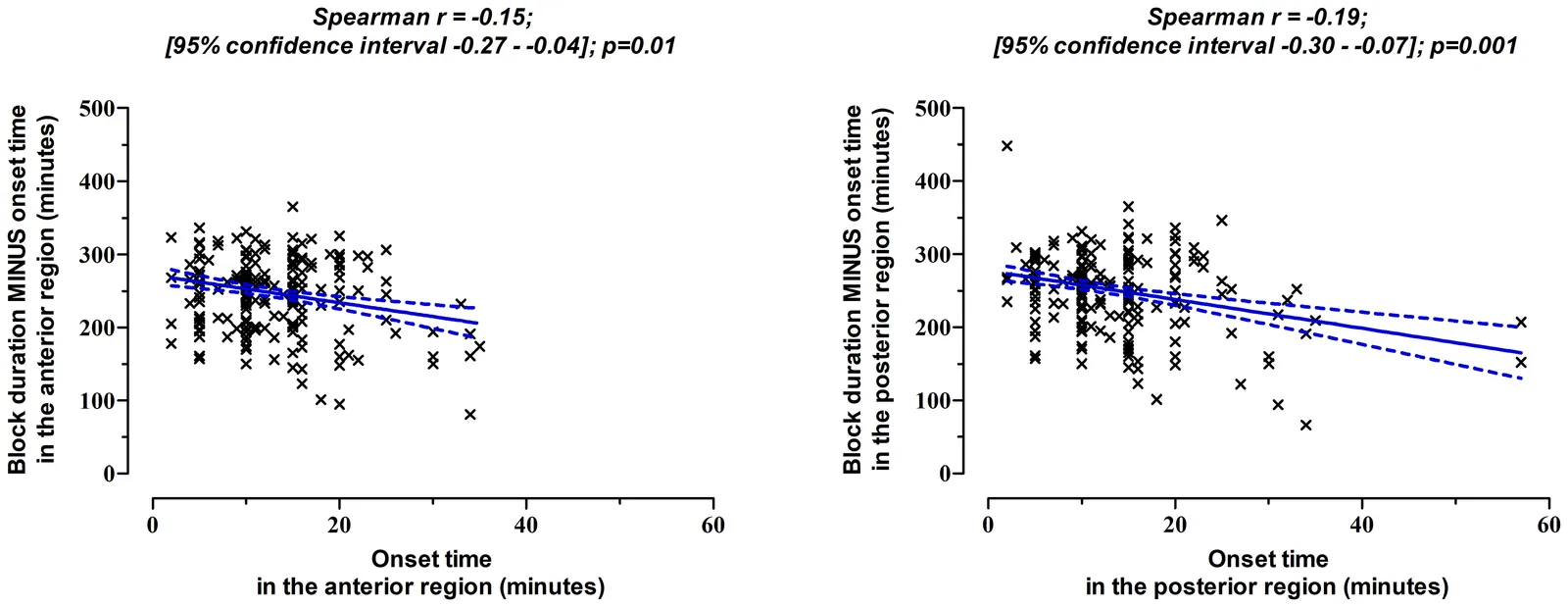
All patients (n = 49). Spearman: Spearman correlation coefficient. The blue dotted line is the 95% confidence interval. The correlation of the block duration minus onset time versus onset time (minutes). Complete loss of sensation = onset time. Complete return of sensation = block duration.

A correlation was found between the first signs and the complete loss of sensation, as well as between the first signs and the full return of sensation (Supplemental Digital Content, S3, https://links.lww.com/AA/F837). Interestingly, there is a negative correlation between block duration (minus onset time) and onset time (Figure 2).

## DISCUSSION

Loss and return of sensation require time. We know from clinical practice that loss of sensation occurs from proximal to distal, as seen in our study. Despite widespread use, the return of sensation related to the anatomic region is unclear.

Local anesthetics bind to the activated and inactivated states of voltage-gated sodium channels, stabilizing them and preventing sodium influx, which inhibits action potential propagation and selectively suppresses high-frequency pain signals.^4^ Therefore, the onset of action depends on the dose (concentration × volume) of the drug and the distance of the injection site to the nerve.^4^ A relatively high volume of 1% mepivacaine was used to ensure sufficient distribution for a reliable block. While concentration and total dose can influence onset and duration,^5^ our approach was intended to achieve consistent coverage across patients.^6^

The onset and duration of the block with 1% mepivacaine in our study (mean onset 12–17 min/mean duration 245–271 minutes) aligned with previous findings (reported in previous studies, mean onset 5–30 minutes/mean duration 256–270 minutes).^5,7^

Previous research has shown that intraneural (subepineural) injection accelerates the onset of nerve block due to the epineurium acting as a diffusion barrier.^8^ If the onset time was short, the block duration was significantly longer in our study. This could be an important reason for the observed negative correlation of onset and block duration.

In our observation, sensation returned faster to the upper arm than to the elbow, forearm, and hand. One possible explanation is that redistribution of the local anesthetic occurs more rapidly than initially assumed, resulting in earlier recovery of proximal regions than predicted by the diffusion-based model. Another hypothesis for our finding could be that if a sufficient local anesthetic concentration in the nerve fiber to be blocked is reached, enough sodium channels are blocked to prohibit an action potential in this nerve fiber for a certain time period. This time period could be interindividual and dependent on the sodium channel expression.^9,10^ Nevertheless, the physiological mechanism remains unclear.

The most important limitation of this study is the small, single population. Furthermore, sensation was evaluated at least every 20 minutes, which may also reduce accuracy.

In conclusion, the loss of sensation was from proximal to distal, as clinically seen. The return of sensation was faster in the upper arm than in the elbow, forearm, and hand. However, the physiological mechanism remains unclear.

## DISCLOSURES

**Conflicts of Interest**: None. **Funding:** The study was funded by departmental resources. **This manuscript was handled by:** Michael J. Barrington, MB BS, FANZCA, PhD.

## Supplementary Material


