# Supplementary material for: Loss and Return of Sensation After Axillary Brachial Plexus Nerve Block: Distally or Proximally?
Source: Anesth Analg. 2026 Jul 23;143(6):1414–7. doi: 10.1213/ANE.0000000000008175 (PMC13747281; doi:10.1213/ANE.0000000000008175)
Supplement: Supplementary file 1 [file ane-143-1414-s001.pdf]

Patientenetikett

Datum: Unterschrift Prüfarzt:

Uhrzeit Anlage des Blockes:

1. Bitte mit Uhrzeit dokumentieren, wann welches Hautareal betroffen war:

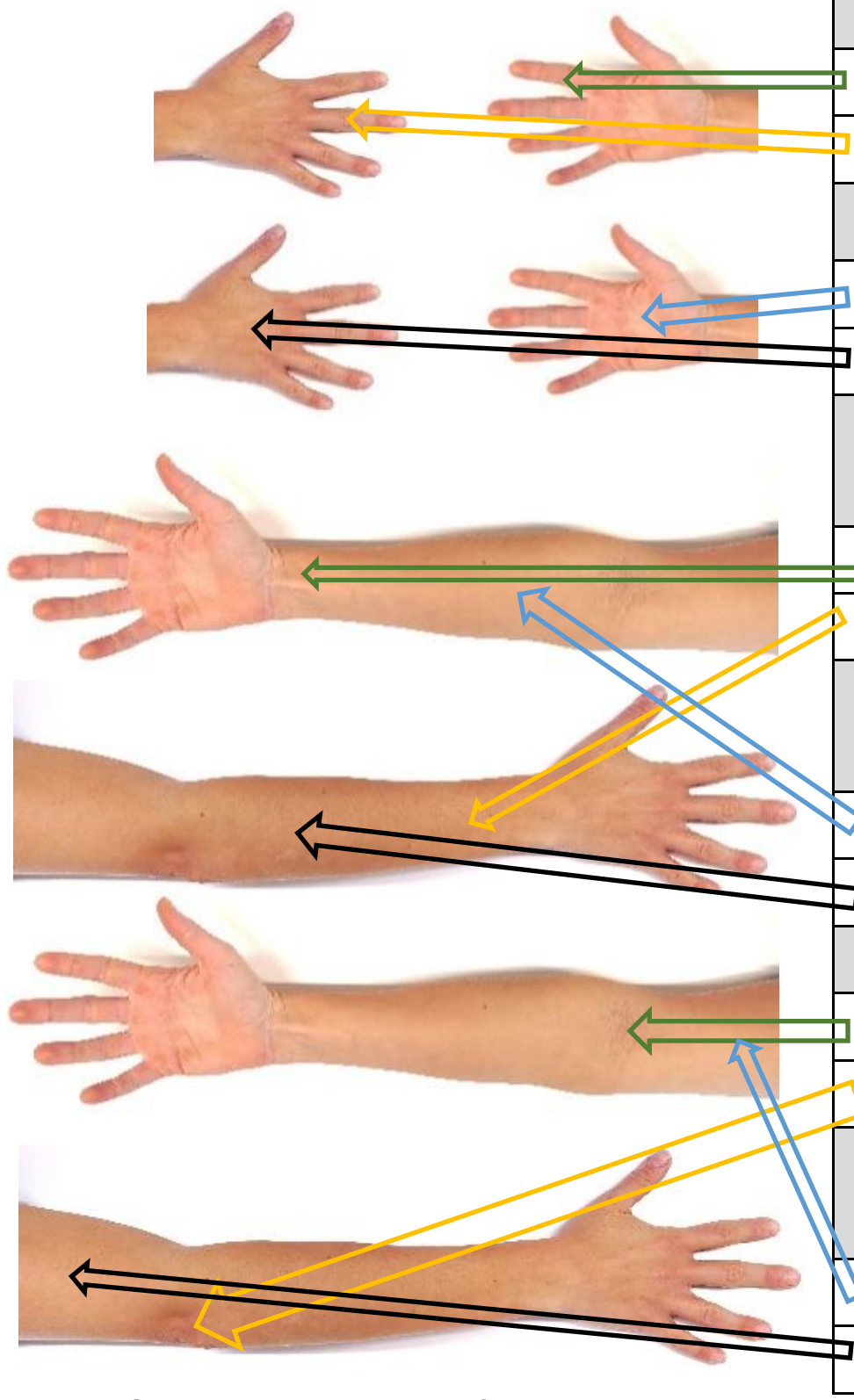

Quelle der Fotos: Eigene Aufnahmen, erstellt in der Universitätsklinik Balgrist.

	Uhrzeit des Einschlafens	
	1. Zeichen	komplett
**FINGER:**		
**Innenfläche (ventral)**		
**Aussenfläche (dorsal)**		
**HAND:**		
**Innenfläche (ventral)**		
**Aussenfläche (dorsal)**		
**UNTERARM UNTEN (= Hand nah)**		
**Innenfläche (ventral)**		
**Aussenfläche (dorsal)**		
**UNTERARM OBEN (= Ellenbogen nah)**		
**Innenfläche (ventral)**		
**Aussenfläche (dorsal)**		
**ELLENBOGEN**		
**Innenfläche (ventral)**		
**Aussenfläche (dorsal)**		
**OBERARM UNTEN (=Ellenbogen nah)**		
**Innenfläche (ventral)**		
**Aussenfläche (dorsal)**		

Patientenetikett

Datum: Unterschrift Prüfarzt:

2. Bitte mit Uhrzeit dokumentieren, wann welches Hautareal zuerst wach wird:

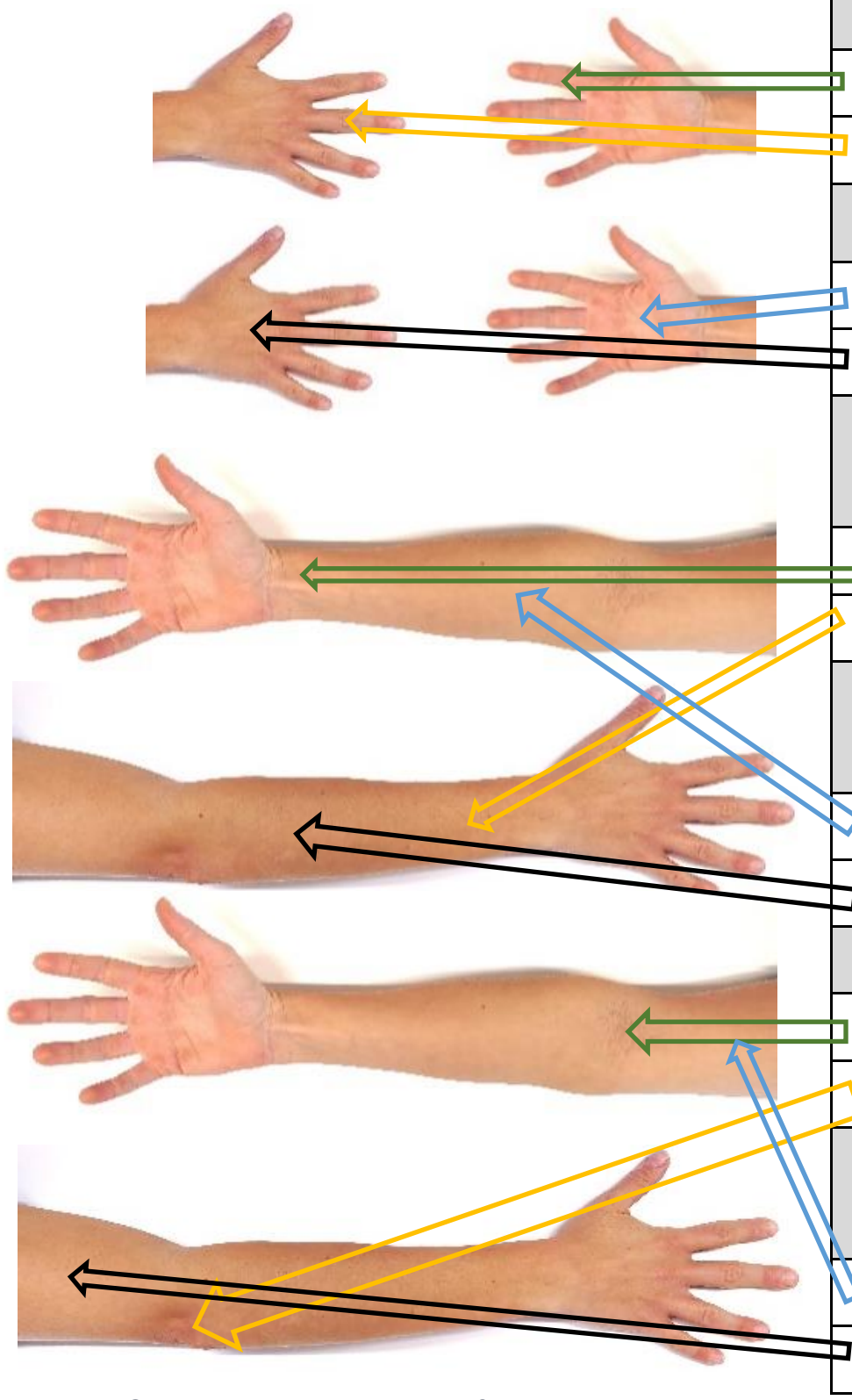

Quelle der Fotos: Eigene Aufnahmen, erstellt in der Universitätsklinik Balgrist.

	Uhrzeit des Erwachens	
	1. Zeichen	komplett
**FINGER:**		
**Innenfläche (ventral)**		
**Aussenfläche (dorsal)**		
**HAND:**		
**Innenfläche (ventral)**		
**Aussenfläche (dorsal)**		
**UNTERARM UNTEN (= Hand nah)**		
**Innenfläche (ventral)**		
**Aussenfläche (dorsal)**		
**UNTERARM OBEN (= Ellenbogen nah)**		
**Innenfläche (ventral)**		
**Aussenfläche (dorsal)**		
**ELLENBOGEN**		
**Innenfläche (ventral)**		
**Aussenfläche (dorsal)**		
**OBERARM UNTEN (=Ellenbogen nah)**		
**Innenfläche (ventral)**		
**Aussenfläche (dorsal)**		

S1: Standardized documentation form with specific instructions

Items	• **Indications of implausible data**
Time of loss of sensation	• Implausible if time of first signs of loss of sensation was after the complete loss of sensation.
Time of the return of sensation	• Implausible if time of first signs of the return of sensation was after the complete return of sensation.
Time of loss and the return of sensation	• Implausible if time of loss of sensation was after the return of sensation.

S2: Proof of plausibility

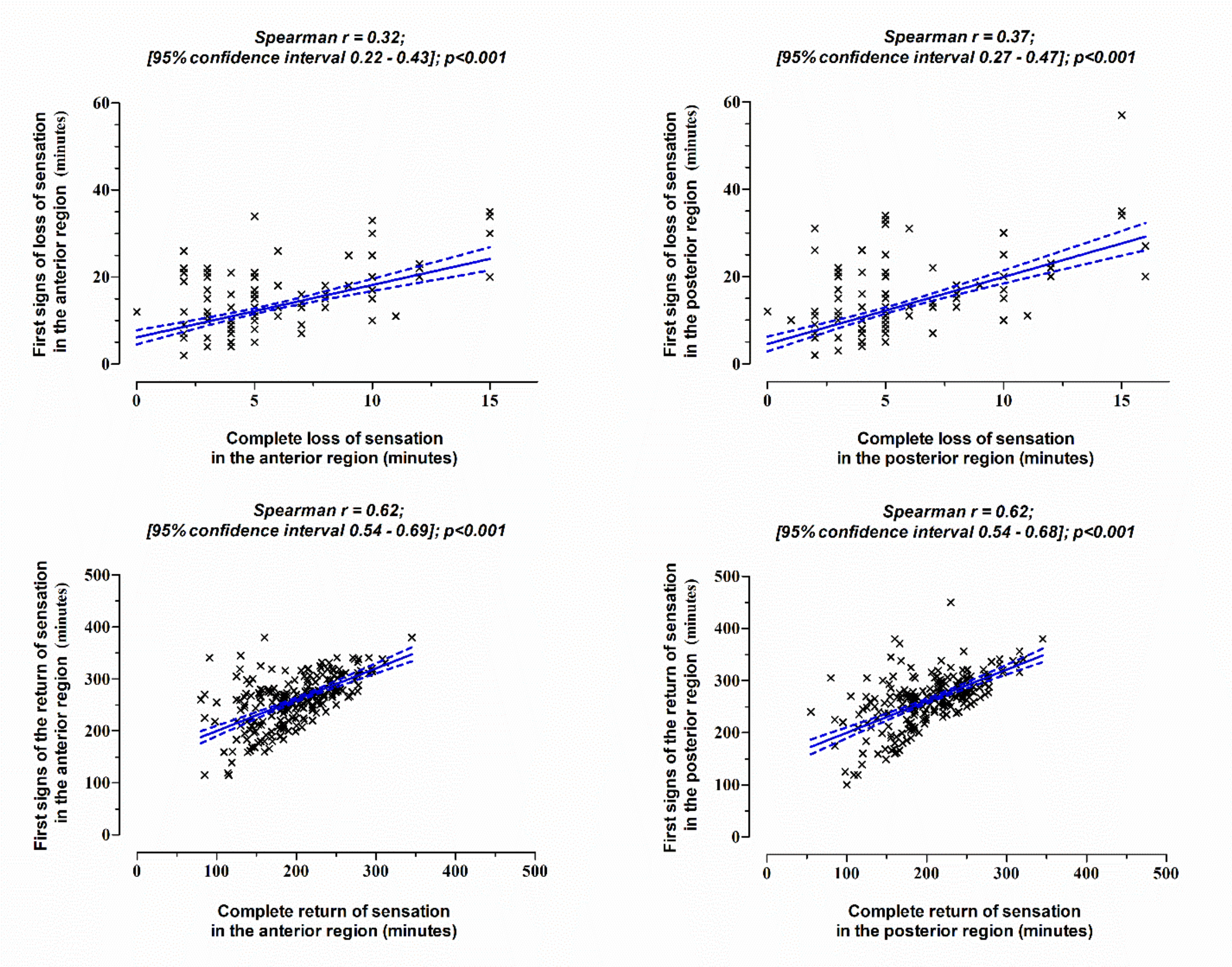

S3: All patients (n=49). Spearman: Spearman correlation coefficient. The blue dotted line is the 95% confidence interval. Upper Figures: The correlation of the first signs versus complete loss of sensation (minutes). Bottom figures: The correlation of the first signs versus complete return of sensation (minutes).

The first signs of loss of sensation: Time between the end of block placement and the first signs of loss of sensation. Complete loss of sensation = onset time: Time between the end of block placement and the complete loss of sensation.

The first sign of the return of sensation: Time between the end of block placement and the first sign of the return of sensation. Complete return of sensation = block duration: Time between the end of block placement and the complete return of sensation.
